# Field Assessment of the Host Range of *Aculus mosoniensis* (Acari: Eriophyidae), a Biological Control Agent of the Tree of Heaven (*Ailanthus altissima*)

**DOI:** 10.3390/insects12070637

**Published:** 2021-07-13

**Authors:** Francesca Marini, Erica Profeta, Biljana Vidović, Radmila Petanović, Enrico de Lillo, Philip Weyl, Hariet L. Hinz, Chandra E. Moffat, Marie-Claude Bon, Tatjana Cvrković, Javid Kashefi, René F. H. Sforza, Massimo Cristofaro

**Affiliations:** 1 Biotechnology and Biological Control Agency (BBCA) Onlus, Via Angelo Signorelli 105, 00123 Rome, Italy; erica.profeta23@gmail.com (E.P.); massimo.cristofaro.cas@enea.it (M.C.); 2 Department of Entomology and Agricultural Zoology, Faculty of Agriculture, University of Belgrade, Nemanjina 6, 11080 Belgrade-Zemun, Serbia; magud@agrif.bg.ac.rs (B.V.); rpetanov@agrif.bg.ac.rs (R.P.); 3 Serbian Academy of Sciences and Arts, Knez Mihailova 35, 11000 Belgrade, Serbia; 4 Department of Plant, Soil and Food Sciences, University of Bari Aldo Moro, Via Amendola, 165/A, 70126 Bari, Italy; enrico.delillo@uniba.it; 5 CABI, Rue des Grillons 1, 2800 Delémont, Switzerland; P.Weyl@cabi.org (P.W.); h.hinz@cabi.org (H.L.H.); 6 Agriculture and Agri-Food Canada, Summerland Research and Development Centre, Summerland, 4200 BC-97, Summerland, BC V0H 1Z0, Canada; chandra.moffat@canada.ca; 7 European Biological Control Laboratory, USDA-ARS, Campus International de Baillarguet, 34980 Montferri-er-sur-Lez, France; mcbon@ars-ebcl.org (M.-C.B.); jkashefi@ars-ebcl.org (J.K.); rsforza@ars-ebcl.org (R.F.H.S.); 8 Institute for Plant Protection and Environment, Department of Plant Pests, Laboratory for Molecular Diagnostics and Laboratory for Applied Entomology, Banatska 33, 11080 Belgrade-Zemun, Serbia; tanjacvrkovic@yahoo.com; 9 ENEA Casaccia, SSPT-BIOAG-PROBIO, Via Anguillarese 301, 00123 Rome, Italy

**Keywords:** tree of heaven, *Ailanthus altissima*, invasive species, biological control, eriophyid mite, *Aculus mosoniensis*, host range

## Abstract

**Simple Summary:**

Tree of heaven is a tree native from Asia, considered a serious weed where ever it occurs outside of its native range. Among the herbivores which attack it is a tiny mite, *Aculus mosoniensis*, which causes serious malformations of the growing tips. In order to determine whether this species is safe to use as a biological control agent in Europe, where the mite has already been recorded, and elsewhere within the invasive range of tree of heaven, host range testing is required. After testing several tree species related to tree of heaven, it was found that this mite was unable to reproduce on any other species except tree of heaven, and importantly there was no damage or sign of feeding on any of the test species. Thus we conclude that this is a safe biological control agent and will help to control this highly invasive tree.

**Abstract:**

Tree of heaven (*Ailanthus altissima*) is a fast-growing deciduous tree native to China, considered a serious invasive species worldwide, with several socio-economic and ecological impacts attributed to it. Chemical and mechanical methods have limited efficacy in its management, and biological controls may offer a suitable and sustainable option. *Aculus mosoniensis* (Ripka) is an eriophyid mite that has been recorded to attack tree of heaven in 13 European countries. This study aims to explore the host range of this mite by exposing 13 plant species, selected either for their phylogenetic and ecological similarity to the target weed or their economic importance. Shortly after inoculation with the mite, we recorded a quick decrease in mite number on all nontarget species and no sign of mite reproduction. Whereas, after just one month, the population of mites on tree of heaven numbered in the thousands, irrespective of the starting population, and included both adults and juveniles. Significantly, we observed evidence of damage due to the mite only on target plants. Due to the specificity, strong impact on the target, and the ability to increase its population to high levels in a relatively short amount of time, we find *A. mosoniensis* to be a very promising candidate for the biological control of tree of heaven.

## 1. Introduction

Tree of heaven, *Ailanthus altissima* (Mill.) Swingle, is one of five known species of the genus *Ailanthus* belonging to the family Simaroubaceae. This deciduous tree is a serious threat to ecosystems in introduced areas and is listed as one of the forty most invasive woody angiosperms from 40 different genera worldwide [1]. Tree of heaven is native to northern and central China [2]. It was introduced to Europe in 1740 for decorative purposes, as the tree was pest and disease free, and into North America in 1781 by accident [3,4], where it is now considered the second most abundant non-native tree species. It has been detected in 48 states [5,6] and occupies over 86,000 ha in southern USA [7,8]; whereas in Canada, it is currently found in a broad range of habitats in southern portions of British Columbia, Ontario and Québec. Similarly, tree of heaven is highly invasive all across Europe [9].

Its expansion on all continents has been facilitated by transfer of seeds and by its ability to grow on poor sites, such as urban areas and fragmented landscapes [10]. Natural disturbances, such as frost or fire, and human-mediated impact by cutting, chopping, or girdling of stems induce a prolific vegetative regeneration by sprouts that may emerge from the root, the root crown, or the stem [2]. Tree of heaven is a dioecious and clonal plant [2,3]. It may produce over 350,000 seeds/year and its seed bank lasts one year under natural conditions, though the seeds may survive several years under laboratory conditions [11]. Seeds can be transported over long distances in the air and up to 450 m high [2].

Tree of heaven has its greatest impacts in anthropic habitats, such as railways and roadsides, causing impressive physical and structural damage to buildings, transport routes and bridges. In the USA, fifty-five percent of every mile of roadway in Virginia contains at least one tree of heaven [12]. Impact in agricultural lands and in rural areas is commonly seen in fields, along roads, fencerows, woodland edges, and forest openings [2,13,14,15]. In western North America (including California, British Columbia, and the southwest of the USA), tree of heaven is most invasive in riparian areas [16], oak woodlands, and open grasslands [17], but also invades other moist habitats, such as along lakeshores. In addition to its direct impacts, tree of heaven hosts high-impact invasive insect pest species, including the spotted lanternfly, *Lycorma delicatula* (Hemiptera: Fulgoridae), an invasive hemipteran from China recently introduced in the USA, which is a pest of several woody tree species [18]. In particular, tree of heaven is used as an overwintering niche for the spotted lanternfly and may be an obligate host for its oviposition [19,20]. Moreover, both in North America and Europe, tree of heaven is a host of the brown marmorated stinkbug, *Halyomorpha halys* (Hemiptera: Pentatomidae) [21], an overwintering household pest and a pest of fruit and vegetable crops. Thus, reducing the populations of tree of heaven would decrease the impact of at least two serious insect pests and improve the native floral diversity in all invaded areas.

Outside its native range, tree of heaven is usually subject to low herbivore pressure, which has been attributed to the chemical composition of its tissues [2]. In fact, it may induce indirect effects on invaded ecosystems as it contains secondary metabolites that make it unpalatable to most phytophagous generalist arthropods, and can be detrimental to native fauna (e.g., amphibians) [22]. The bark of roots and other plant parts contain allelopathic compounds that are also toxic to numerous woody and herbaceous plant species [23]. The quassinoid compound ailanthone was identified as the most effective toxic component, exhibiting an inhibitory activity against several weed species [2,23]. Kowarik and Samuel [2] reported that toxicity is higher in young leaflets just after emergence and then decreases with leaf age. Greer and Aldrich [24] demonstrated, in germination experiments, that the toxicity of tree of heaven is greater in juveniles younger than two years old than in older aged classes.

Besides sexual reproduction, tree of heaven is also capable of clonal reproduction, with propagative roots producing new ramets [25]. For this reason, conventional agronomic control strategies, combining cutting and systemic herbicide applications show limited impact in defeating tree of heaven, thus an environmentally friendly alternate control management would be beneficial. In fact, tree of heaven is currently in the top 20 environmental weeds identified as targets of classical biological control in Europe [26].

In the search for phytophagous arthropods, the U.S. Department of Agriculture (USDA) carried out surveys in China during the early 2000s and identified nearly 60 natural enemies associated with tree of heaven that can be potential candidates as biological control agents [2,4,27].

Among the arthropods, a special emphasis has been placed on the weevil species *Eucryptorrhynchus brandti* (Harold) and *E. chinensis* (Olivier) (Coleoptera: Curculionidae) [4,28,29]. Both species are native to China [30] and prefer to colonize older and taller trees [31]. Adults feed on leaf buds and females deposit eggs under the bark [28], where larvae play a very important role in damaging the tree [4]. The last petition for the field release of *E. brandti* was submitted in the USA in 2018, but in April 2019 the U.S. Department of Agriculture, Animal Plant Health Inspection Service, Technical Advisory Group (USDA APHIS TAG) did not recommend its release [32]. In response, additional testing is currently ongoing [33].

Parallel studies focused on eriophyid mites (Acari: Eriophyoidea), well known for their high degree of host-specificity [34] and for their impact on their associated host, despite their extremely small size [35]. There are four eriophyid species associated with tree of heaven: *Aculops ailanthi* Lin, Jin and Kuang, *Aculus altissimae* Xue and Hong, *Aculops taihangensis* Hong and Xue, and *Aculus mosoniensis* (Ripka) [4,36]. All species, except *A. mosoniensis*, have been recorded for the first time in China. *Aculus mosoniensis* and *Ac. ailanthi* are the only species found outside of the native range of the plant, i.e., in the USA (first record in 2007 [37]) and Europe (first record in 2014 [38]), respectively. However, based on morphological observations [36] and molecular comparison [39], it has been realized that *A. mosoniensis* and *Ac. taihangensis* are synonymous [40]. At the same time, according to J. Amrine, *A. altissimae* may be a junior synonym of *Ac. ailanthi*, because morphological differences between the species are slight and may be attributable to procedural artifacts [41].

*Aculus mosoniensis* has been recorded in 13 European countries, including Albania, Austria, Bulgaria, Croatia, Greece, Hungary, Italy, Macedonia, Montenegro, Romania, Serbia, Slovenia [42], and, more recently, also France [43]. This mite forms dense populations mainly on the underside of the leaflets of compound leaves, but in the case of high infestations, mites can also be found on the upper surface and on all green organs, including stems. The lamina of infested leaflets is narrow, deformed, and twisted with edges folded or rolled upward with respect to the main vein of the leaflets. On heavily infested plants, leaflets can assume a light pale/yellowish aspect or be lost prematurely. Moreover, drying and necrosis of the apical parts of stems and of young plants have also been observed [36]. The considerable impact that this mite can have on its host has led a team of scientists from several international institutions to focus their attention on the assessment of *A. mosoniensis* as a potential biological control agent against tree of heaven.

The main aim of the current study was to evaluate the host range of *A. mosoniensis*, as a prospective candidate for the biological control of tree of heaven. Specifically, 13 plant species were tested with the mite under open-field conditions in 2019 and 2020.

## 2. Materials and Methods

### 2.1. Host Range Study

Test plant species and varieties to evaluate the host-plant specificity of a potential biological control agent are usually selected according to the centrifugal phylogenetic method [44], which assumes that plant species more closely related to the target weed are more likely to be subject to attack than more distantly related plant species. Since there are no species in the family of Simaroubaceae present in Europe, nontarget species were selected among those belonging to the same order of the target (i.e., Sapindales). In addition, plant species of economic value and ecological similarity to tree of heaven were selected on the basis of their presence in Western Europe and an overlapping distribution with *A. altissima*. Finally, the choice of the species tested was contingent upon their availability from Italian nurseries. A total of 14 nontarget species were chosen to be exposed to *A. mosoniensis* under natural field conditions by two open-field tests carried out at BBCA (Biotechnology and Biological Control Agency ONLUS) in Rome (Italy) in 2019 and 2020, respectively (Table 1).

**Figure 1:**
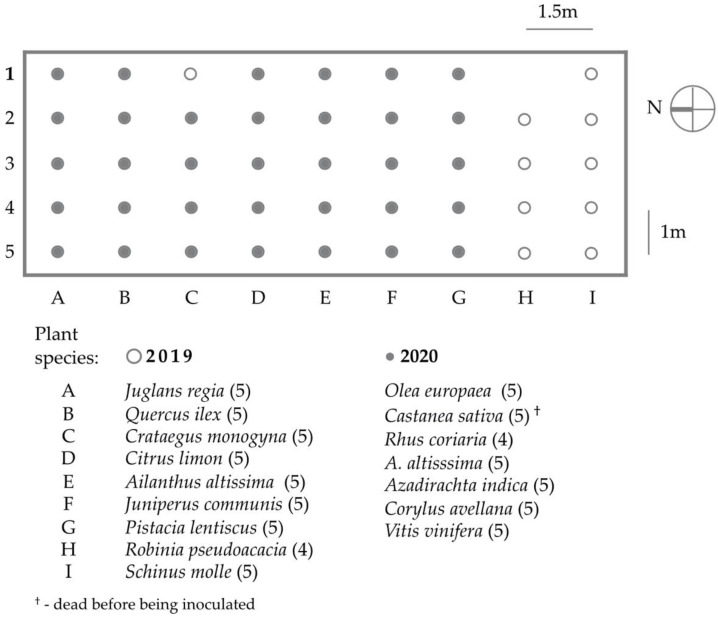
Map of the garden plot setup for target, *Ailanthus altissima* and nontarget plants inoculated with *Aculus mosoniensis* during host-specificity tests performed in 2019 and 2020, respectively. The number of replicates for each species is reported in brackets.

All plants were bought in a nursery, except *Azadirachta indica*, which was grown from commercial seeds in the spring of the year of testing (2020). In both experiments, the plants were arranged in a garden plot organized as a grid, i.e., one species per column and one replicate per row, with plants of the same species located ~1 m apart and those of differing species ~1.5 m, and tree of heaven plants located in the middle (Figure 1). About one week prior to being inoculated with mites, plants were transplanted into soil, in order to best simulate natural conditions and prevent any potential stress caused by maintaining tree species in pots. Only tree of heaven plants were kept in pots, which were set in holes in the ground to prevent them from sprouting into the surrounding area. The ground of the garden plot was covered with plastic foil, to prevent the growth of weeds, and the plants were watered as needed. All plants tested in 2019 were removed from the field garden in the fall of the same year and the soil was prepared for the planting of the plants selected for testing in 2020. In 2020, a new group of tree of heaven plants was used.

In 2019, the garden plot was set up on 23 May. On 30 May, leaves of tree of heaven infested with *A. mosoniensis* were collected from a natural site (Cesano, Rome, Italy; 42°03′50″ N, 12°19′44″ E, 240 m asl) and held at 4 °C until they were used. Each leaf was checked under a stereo microscope at 20× magnification and only those infested with at least 15 living (i.e., motile) mites were selected for use. In the evening of the same day, an infested leaf was attached by a clip to a couple of young leaves of each plant, to allow the voluntary and active movement of mites from infested leaves (inocula) to plants [45]. The inocula were removed once they became completely dry (i.e., 36–48 h later). On the inoculation day, all plants were small (i.e., *A. altissima*, *Crataegus monogyna*, *Juniperus communis,* and *Pistacia lentiscus* plants were about 50 cm tall, while *Citrus limon*, *Juglans regia,* and *Quercus ilex* plants were approximately 100 cm, and *Robinia pseudoacacia* and *Schinus mole* plants were roughly 150 cm), and in the vegetative stage (no flowers or fruits) with growing tips. From 2 to 4 July, about 34 days after the inoculation, a cutting was collected from each plant. Cuttings were made above and as close to the inoculation points as possible. Mites were extracted from leaves, excluding stems and any woody part of the cutting samples, and the wet weight of the processed leaves was measured using a precision balance (PCE-BS 300, PCE Instruments, Meschede, Germany). The extractions were carried out following the protocol described by Monfreda et al. [46], and the mites obtained were transferred to a 4.7 cm diameter cellulose nitrate filter with 20 μ mesh openings (Sartorius). Mites were counted under a stereo-microscope at 20× magnification and then stored in 70% ethanol, separating live and dead mites (i.e., motile and non-motile), and subsequently identified based on morphology (i.e., species, stage and gender—adult females and males, and juveniles—were determined). The same procedure was repeated from 15–16 and 29–30 July, about 47, and 61 days post-inoculation, respectively.

In 2020, due to the restrictions enforced by COVID-19, it was only possible to set up the open-field test late in the season, i.e., on 25 June. However, the plants were still small, (i.e., similarly to 2019, all plants were roughly 50 cm tall, with the exception of *Olea europaea* and *Vitis vinifera* plants, which were about 100 cm and 150 cm, respectively) and some species (i.e., *A. altissima*, *Az. indica*, and *Rhus coriaria*) were still in the vegetative stage, with growing tips. Leaves of tree of heaven infested with *A. mosoniensis* were collected on 30 June from the same location as in 2019 and the mites were inoculated on plants following the protocol described above. However, since it was late in the season, the number of mites per leaf was in the thousands, and it was impossible to count all or select only some of them. Thus, in an effort to standardize the experimental set up, only infested leaves of similar size were used (about 6.5 × 2.0 cm). The number of live mites inoculated per plant was estimated by performing extractions of the mites from a small sample of infested leaves (N = 6) of similar size to those used as inocula. The extractions were carried out as described above; for samples that had a large number of mites, only the mites in 11 diagonal cells of the cellulose nitrate filter were counted and, in order to estimate the total number of mites, the number obtained was multiplied by the ratio of the area of the filter to that of the cells considered. From 4 to 6 August and from 31 August to 2 September, i.e., about 36, and 63 days after inoculation, a cutting, made as close to the inoculation points as possible, was collected from each plant, whereupon mites were extracted, counted, and identified following the same protocol applied in 2019. During the first sampling, due to a large number of mites found, only the number of live (i.e., motile) mites was determined.

### 2.2. Molecular Identification of Aculus Mosoniensis

A sample (N = 4) of the mite population collected from Cesano (Rome, Italy) and used for the inoculation of the plants tested during both field experiments was brushed from a leaf under a stereomicroscope (magnification 100×), preserved in 96% ethanol in collecting tubes, and stored at 4 °C until further DNA analysis at the Department of Plant Pests, Institute for Plant Protection and Environment (IPPE, Belgrade-Zemun, Serbia). Genomic DNA was extracted from several pools of 5–10 specimens using QIAGEN DNeasy Blood & Tissue Kit (QIAGEN, Hilden, Germany), according to the manufacturer’s instructions, with modifications based on [47] and following Skoracka et al.’s [48] recommendations to eliminate the concern about possible multiple operational taxonomic units within DNA samples extracted from multiple specimens. Residual DNAs are archived at IPPE. The barcode region of the mitochondrial cytochrome oxidase subunit I gene (mtCOI) sequence was amplified in a 25 μL final volume, using the pair of primers LCO1490/HCO2198 [49]. Polymerase chain reactions (PCR) were performed using 1x high yield reaction buffer with MgCl_2_, 2.5 mM MgCl_2_, and 0.6 mM of each dNTP, 0.5 μM of each primer, 1 unit of KAPATaq DNA polymerase (Kapabiosystems), and 5.0 μL of diluted template DNA. The thermal cycling program was carried out in a Mastercycler ep gradient S (Eppendorf, Hamburg, Germany) applying the following thermal steps: 95 °C for 5 min (initial denaturation), 40 cycles at 94 °C for 1 min, 1 min at 48 °C (annealing), 1 min 30 s at 72 °C, and a final extension at 72 °C for 7 min. The amplified fragments were purified using the QIAquick PCR purification Kit (QIAGEN, Hilden, Germany) according to the manufacturer’s instructions, and sequenced in both directions with the same primer pairs as in the initial PCR procedure on automated equipment by Macrogen (Seoul, Korea) for IPPE. The sequences were manually edited using FinchTV software v.1.4.0 [50], and were translated into amino acids to check for the absence of any stop codon in this protein coding gene. To confirm the taxonomy of morphologically identified specimens, this sequence was compared with sequences present in GenBank by similarity search using the Basic Local Alignment Search Tool [51].

### 2.3. Statistical Analysis

Statistical analyses were carried out using RStudio software Version 1.2.5042 [52]. All parameters were tested for normality by Shapiro–Wilk tests and the homogeneity of error variances by Bartlett tests; the data were analyzed with a parametric or non-parametric test when assumptions were not fulfilled. Due to the differing leaf shapes of the plant species tested, the wet weight of the leaves collected and processed was used to estimate the number of *A. mosoniensis* (live or dead) per gram of infested plant, making the samples comparable. Differences in the densities thus determined were tested by Kruskal–Wallis rank sum tests, followed by pairwise comparisons using the Wilcoxon rank sum test. The same approach was used in testing for differences in the number of live mites per gram of plant biomass collected from tree of heaven on various sampling dates (i.e., at 34, 47, and 61 days post-inoculation in 2019), as well as to compare the densities of live females, males, and juveniles. Finally, the differences in the densities of live *A. mosoniensis* on *A. altissima* at about one and two months from the inoculation between the two field experiments were tested by the Welch test.

## 3. Results

**Figure 2:**
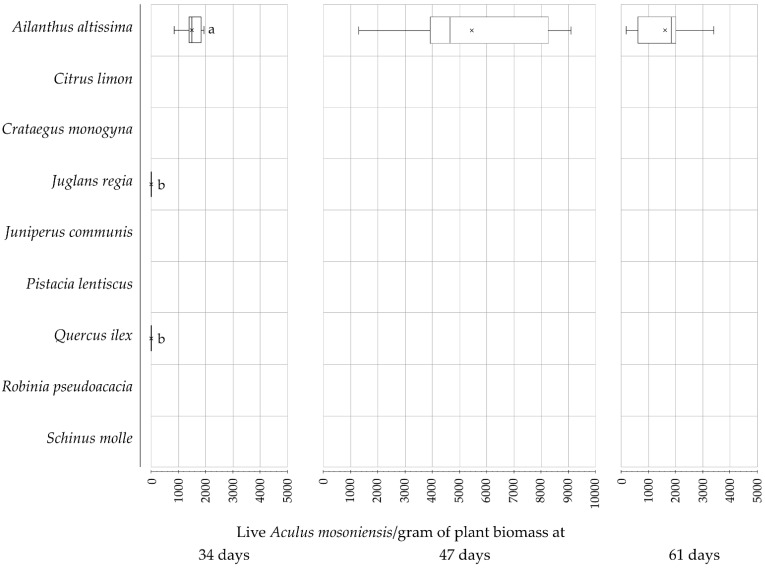
Number of live *Aculus mosoniensis* per gram of plant biomass collected from the plant species tested at 34, 47, and 61 days post-inoculation, respectively, in the field experiment performed 2019.The boxes represent the first to third quartile range with the median indicated by a line across the box and the mean by crosses. Bars flanked by different letters differ significantly at *p* = 0.05.

Average numbers of live *A. mosoniensis* per gram of plant biomass collected from the plant species tested at different sampling dates (i.e., 34, 47, and 61 days post-inoculation, respectively) in 2019 are presented in Figure 2 and in Appendix A, Table A1. At 34 days from the inoculation, mites were found on both target and nontarget plants; however, live mites were collected only from *A. altissima*, *J. regia,* and *Q. ilex* plants. The densities of live mites on *J. regia* and *Q. ilex* were similar to each other (0.6 ± 0.3 [SE] and 1.1 ± 0.4, respectively), and were both over 1000 times lower than on tree of heaven (1486.8 ± 194.3 [SE]) (Kruskal–Wallis rank sum test, $\chi^{2\;}$= 33.203, df = 8, *p*-value = 5.661 × 10^−5^; Wilcoxon rank sum test, *p* < 0.05). In particular, only a few live mites were collected from *J. regia* and *Q. ilex*, i.e., 11 (plus 25 dead) and 8 (plus 8 dead) individuals, respectively. Live juveniles were found only on *Q. ilex* and *A. altissima*, and they were 37.5% and 6.6% of the live mites collected (i.e., 3 and 610 individuals), respectively (Appendix A, Table A1). At 47 and 61days post-inoculation, no live mites were found on any of the nontarget species tested, whereas the density of live *A. mosoniensis* on *A. altissima* was 5441.4 (±1437.4 [SE]) and 1588.3 (±567.7 [SE]), respectively.

**Figure 3:**
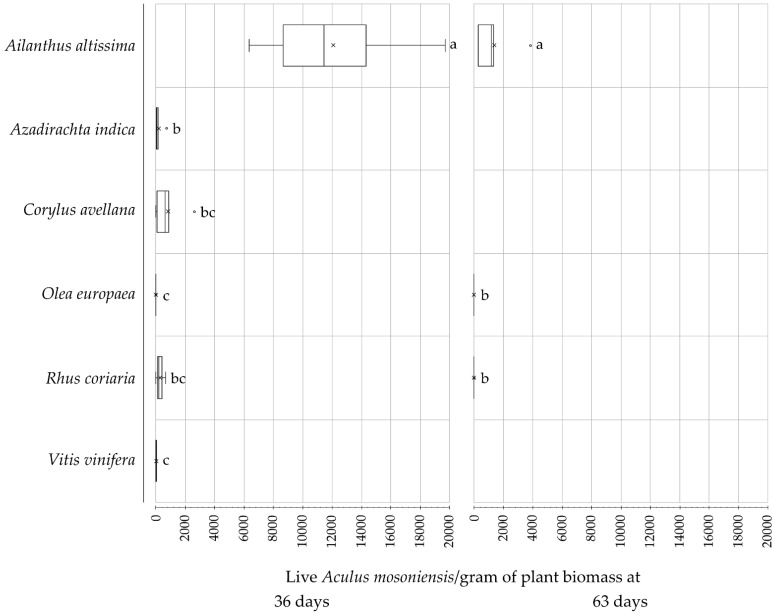
Number of live *Aculus mosoniensis* per gram of plant biomass collected from the plant species tested at 36 and 63 days post-inoculation, respectively, in the field experiment performed 2020. The boxes represent the first to third quartile range with the median indicated by a line across the box, and the mean by crosses. Outliers are indicated by empty dot. Bars flanked by different letters differ significantly at *p* = 0.05.

In 2020, plants of *C. sativa* died immediately after being transplanted and before being inoculated, therefore they were not tested and are not mentioned in tables and figures hereafter. A high number of live mites (6755.0 ± 1513.1 [SE]) were inoculated on each plant. Average numbers of live *A. mosoniensis* per gram of plant biomass collected from the plant species at 36 and 63 days post-inoculation, respectively, are presented in Figure 3 and in Appendix A, Table A2. At 36 days from inoculation, *A. mosoniensis* was found on both target and nontarget plants. Live mites were collected from all species, but not from all nontarget plants inoculated, and the densities of live mites recorded on them were significantly different among species (Kruskal-Wallis rank sum test, $\chi^{2}$= 19.24, df = 5, *p*-value = 0.001734; Wilcoxon rank sum test, *p*-value < 0.03). In particular, the density of live mites on *A. altissima* (12,096.2 ± 2324.6 [SE]) was higher than on any of the nontarget species tested. At the end of the experiment, i.e., 63 days post-inoculation, live mites were still found on tree of heaven and on two nontarget species, *O. europaea* and *R. coriaria*. The densities of live mites on *O. europaea* and *R. coriaria* did not differ significantly from each other (0.2 ± 0.1 [SE] and 0.5 ± 0.5, respectively), and were both over 1000 times lower than on tree of heaven (1378.5 ± 657.7 [SE]) (Kruskal-Wallis rank sum test, $\chi^{2}$= 21.423, df = 5, *p*-value = 0.0006736; Wilcoxon rank sum test, *p* < 0.05). In particular, only few mites were collected from *O. europaea* and *R. coriaria* (3 live and 1 dead mite from *O. europaea*, and 1 live mite from *R. coriaria*). No live juveniles were found on any nontarget plants at either sampling date, whereas 0.2 and 4.2% of live mites collected from tree of heaven at 36 and 63 days post-inoculation were identified as juveniles (i.e., 102 and 223) (Appendix A, Table A2).

**Figure 4:**
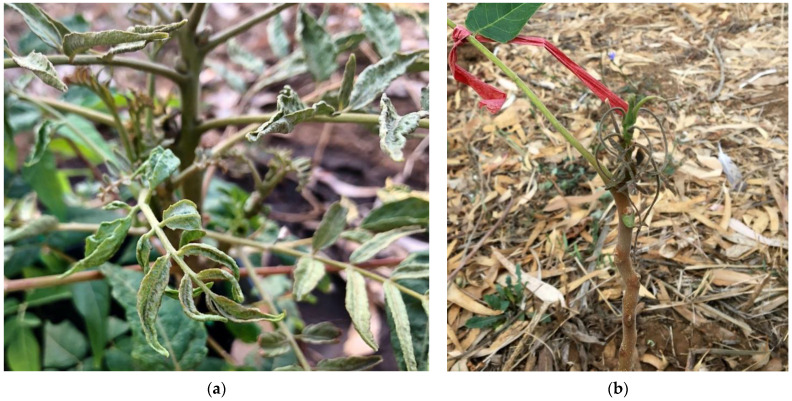
Symptoms of *Aculus mosoniensis* on *Ailanthus altissima*: (**a**) leaves strongly deformed 47 days post-inoculation in 2019; (**b**) dried and necrotic apical part 63 days after inoculation in 2020.

No damage by mites was noted on any of the nontarget plants in 2019 or in 2020, whereas extensive symptoms of feeding damage were already apparent on tree of heaven plants about one month from the inoculation, and a couple of weeks later the leaves were strongly deformed (Figure 4a). At the end of both experiments, *A. altissima* plants were stunted, had lost most of their leaves and drying and necrosis of the apical parts of stems was observed (Figure 4b).

In 2019, the average number of live mites (i.e., adults and juveniles) per gram of plant biomass collected from *A. altissima* did not differ during the experiment, even though a general trend was observed (Figure 2, Table 2). The density of live *A. mosoniensis* increased during the first 47 days after the inoculation, and then it decreased during the last two weeks of the experiment to a density similar to that recorded after 34 days post-inoculation. The same pattern was observed for the density of live adult females and males (Table 2). In particular, the average number of live *A. mosoniensis* males per gram of plant biomass of *A. altissima* at 47 days post-inoculation was different from the density recorded respectively at 34 and 61 days (Kruskal-Wallis rank sum test, $\chi^{2}$= 6.86, df = 2, *p*-value = 0.0323; pairwise comparison using Wilcoxon rank sum test as post-hoc, *p* < 0.05), which on the contrary were similar on each other (Table 2). The density of live juveniles appeared to progressively decrease during the experiment, although the difference between the three sampling dates was not significant (Table 2).

**Figure 5:**
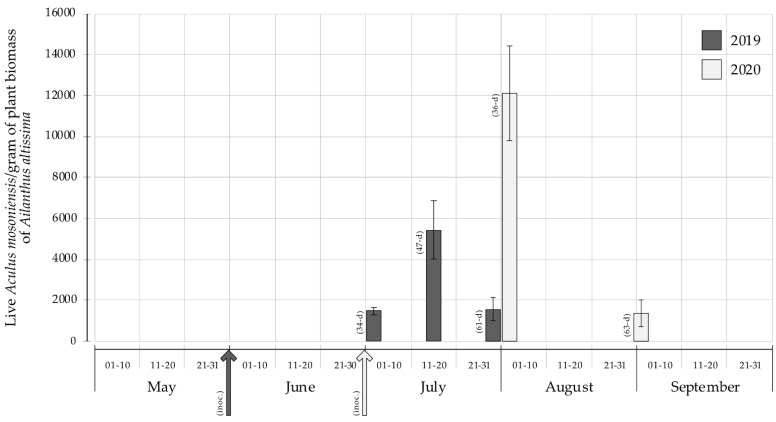
Number of live *Aculus mosoniensis* per gram of plant biomass collected from *Ailanthus altissima* (mean ± SE) through the season in field experiments performed in 2019 and 2020, respectively. The arrows represent the inoculation day (inoc.), whereas days after inoculation are indicated in brackets flanking the bars (34, 47 and 61 days in 2019; and 36 and 63 days in 2020).

The mite population dynamics on tree of heaven plants through both the 2019 and 2020 experiments are presented in Figure 5. The density of live *A. mosoniensis* on tree of heaven at about one month from the inoculation (i.e., 34 and 36 days in 2019 and 2020, respectively) was lower in 2019 than in 2020 (1486.8 ± 194.3 [SE] vs. 12,096.2 ± 2324.6: Welch test, *t* = −4.548, df = 4.0559, *p*-value = 0.0101). However, at the end of the experiment (i.e., 61 and 63 days post-inoculation in 2019 and 2020, respectively), the density of live mites on *A. altissima* did not differ (1588.3 ± 567.7 [SE] vs. 1378.5 ± 657.7), despite their different number of mites inoculated at the beginning.

Finally, barcode-compliant sequences were successfully obtained for each pool of mites sampled, and none of the characteristic evidence of NUMTs was present in the sequences obtained. As all sequences were similar, only one barcode sequence was submitted to the GenBank database, on 7 June 2021 (GenBank Accession number MZ398134). This sequence was 100% identical to the sequence (GenBank Accession number MW892618.1) also obtained from samples collected in Cesano (Rome, Italy), and recently deposited by Kashefi et al. [43].

## 4. Discussion

The host range of the eriophyid mite *A. mosoniensis* has been evaluated by testing 13 nontarget species in two field experiments. During both experiments, the density of live mites on nontarget species quickly decreased; however, a few of them were able to persist on some nontarget plants up to about one or two months. Since *A. mosoniensis* is present in the Lazio region of Italy, where the field bioassays took place, it might be possible that the few *A. mosoniensis* found on the nontarget plants entered the test from the surrounding area. This scenario is not unusual [53,54,55,56] or unrealistic, especially considering that eriophyid mites disperse mainly by wind [57,58,59]. Although mites actively initiate dispersal, once aloft, they have little or no control over where they land. Eriophyid mite dispersal is hence a passive process resulting in their random deposition throughout the environment [60]. However, given the low numbers of live *A. mosoniensis* collected from only a few nontarget plants, which were not the closest located to tree of heaven plants (except for *R. coriaria* plants), we assume these individuals were of those originally inoculated that had survived since the inoculation date.

Although the life expectancy of mites is poorly known, non-diapausing females (i.e., protogyne morphs) have been reported to survive 4–5 weeks [61]. However, the lifespan of some species under cool conditions can be even longer, especially if they stay hydrated and obtain some nutrition. For example, Valenzano et al. [62] observed that some species in water droplets can survive from 1 to 11 days at 25 °C, and 1 to 7 weeks at 5 °C (depending on species and morph). Other species have also proven to be long-lived on unsuitable substrates, such as *Aceria tulipae* (Keifer) (i.e., *Aceria tosichella* Keifer), which survived at least 80 days on an artificial substrate [63], or *Aceria salsolae* de Lillo and Sobhian, which persisted on the nontarget species *Atriplex coronata* up to approximately 55 days under field conditions [64]. Therefore, the presence of a few adults, and no juveniles, of *A. mosoniensis* on some nontarget species after more than one or two months can probably be explained by the ability of this mite to persist for long periods on hosts otherwise not completely suitable.

It is important to note that after about two months from the beginning of both experiments, juveniles were found only on tree of heaven plants, suggesting that *A. mosoniensis* was only able to reproduce on tree of heaven and not on any of the 13 nontarget species tested. Similarly, *A. salsolae* did not reproduce on *A. coronata* under field conditions [64], and *A. tulipae* (i.e., *A. tosichella*) was observed to oviposit only once it was transferred to wheat plants [63], suggesting that reproduction may occur only on the most suitable host plants.

In addition, none of the nontarget plants showed any kind of damage due to the mite. This is not too surprising, considering the very low number of individuals of *A. mosoniensis* recorded on nontarget species and its lack of reproduction. The level of damage that an arthropod can cause to a plant usually depends on the number of individuals attacking the plant [65]. Therefore, it is unlikely that *A. mosoniensis* posed a risk to any of the nontarget species tested. In contrast, the extensive symptoms already recorded on tree of heaven at about one month after the inoculation confirmed what had already been observed by de Lillo et al. [36] (i.e., leaflets strongly deformed or lost prematurely, and drying and necrosis of the apical parts of stems and of young plants).

Particularly relevant is the damage recorded on the apical part of the stems of the tree of heaven plants tested. According to Krussmann et al. [11], mass releases of either a pathogen or a herbivore would, in a few years, eliminate large stands of tree of heaven. However, single trees and isolated stands would still need to be removed by chemical or mechanical means. As a result, a host-specific herbivore that could both tolerate and bypass toxic compounds, and efficiently damage the sprouts and young plants of tree of heaven, would be useful for control of this tree on a large scale. For example, it might be used in concert with other herbivores preferring taller and older plants, such as the weevil *E. brandti*, or in combination with conventional agronomic control strategies.

The trend observed in the *A. mosoniensis* population dynamic during the experiment carried out in 2019 has also been reported for other mite species. In particular, the population dynamics of some phytophagous mites have been observed to follow both temperature and plant phenology patterns. For example, the density of *Tegolophus guavae* (Boczek), a vagrant mite attacking guava trees, was recorded to grow as leaf nutrients and temperature increased [66]. Another mite belonging to the same genus, the olive rust mite *T. hassani* (Keifer), was observed to reach its highest population densities on leaves between the end of the spring and early summer [67].

Finally, the comparison of the *A. mosoniensis* densities recorded on *A. altissima* at the end of both experiments showed that despite the two experiments differing in the time of initiation and in the number of mites inoculated, the density of mites on tree of heaven after approximately two months was similar. This might be explained by the fact that at the end of both experiments, *A. altissima* plants were strongly compromised by the presence of *A. mosoniensis* and only a few leaves were still available for the mites.

In conclusion, *A. mosoniensis* is not able to multiply on any of the nontarget species tested, and none of them appear to be potential hosts of this mite. Furthermore, the mite did not negatively impact any of the nontarget species tested when it did occur on them, even when transferring an extremely high number of mites, as in 2020. Although a complete evaluation of the host specificity of the mite under open field conditions (i.e., its ecological host range) also requires the testing of plant species in the family Simaroubaceae, *A. mosoniensis* appears to be restricted to tree of heaven, *A. altissima*, at least in the European context under consideration. A test plant list for North America is currently being developed; however, it should not be ignored that many of the nontarget species tested, especially those selected for their economic value, also have relevance in several other countries. Therefore, *A. mosoniensis* may be a promising candidate for the biological control of tree of heaven not only in the European context, but also in other regions with similar climate. Finally, as *A. mosoniensis* is already present in Europe, it would not be released as an exotic species, but would rather be redistributed and/or used in an augmentative approach for the control of tree of heaven.

## Figures and Tables

**Table 1:** Nontarget species selected to be tested under open-field conditions. Plant species are listed according to their phylogenetic or ecological association to tree of heaven, *Ailanthus altissima*, and their economic value.

Order	Family	Species	Common Name
Phylogenetically related			
Sapindales	Anacardiaceae	*Pistacia lentiscus* L.	mastic tree
		*Rhus coriaria* L.	Sicilian sumac
		*Schinus molle* L.	false pepper tree
	Meliaceae	*Azadirachta indica* A. Juss.	neem tree
	Rosaceae	*Crataegus monogyna* Jacq.	hawthorn
	Rutaceae	*Citrus limon* (L.) Osbeck	lemon
Ecologically associated			
Fagales	Fagaceae	*Robinia pseudoacacia* L.	black locust
Pinales	Cupressaceae	*Juniperus communis* L.	common juniper
Economically important			
Fagales	Betulaceae	*Corylus avellana* L.	hazel
	Fagaceae	*Castanea sativa* Mill.	chestnut
		*Quercus ilex* L.	holm oak
	Juglandaceae	*Juglans regia* L.	walnut
Lamiales	Oleaceae	*Olea europaea* L.	European olive
Vitales	Vitaceae	*Vitis vinifera* L.	grapevine

**Table 2:** Number of live *Aculus mosoniensis* (total, i.e., adults and juveniles), and live adult females, and males, and juveniles per gram of plant biomass collected from *Ailanthus altissima* (mean ± SE) at 34, 47, and 61 days post-inoculation, respectively, in the field experiment performed in 2019. Values followed by letters in brackets differ significantly at *p* = 0.05.

Days Post-Inoculation	Live *Aculus mosoniensis* Per Gram of Plant Biomass			
	Total	Females	Males	Juveniles
34	1486.8 ± 194.3	630.3 ± 77.3	758.0 ± 103.2 (a)	98.4 ± 33.3
47	5441.4 ± 1437.4	2983.7 ± 1065.4	2413.9 ± 579.9 (b)	43.7 ± 28.9
61	1588.3 ± 567.7	1027.9 ± 461.0	554.4 ± 206.4 (a)	6.0 ± 6.0

## Data Availability

The data presented in this study are available on request from the corresponding author.

## Appendix A

**Table A1:** Number of replicates for each plant species [N]; percentage of plants infested with live *Aculus mosoniensis*; number of live and dead *A. mosoniensis* per gram of plant biomass (mean ± SE) collected from each plant species tested; proportion of live and dead *A. mosoniensis*, and of adult females and males, and juveniles among the live *A. mosoniensis* collected from each plant species tested, at different sampling dates (i.e., 34, 47, and 61 days post-inoculation, respectively) in 2019.

Plant Species [N]	Year	Plants with Live *Aculus mosoniensis*	*Aculus mosoniensis* Per Gram of Plant Biomass		Proportion of *Aculus mosoniensis*	
			Live	Dead	Live [Females, Males, Juveniles]	Dead
**34 days post-inoculation**						
*Ailanthus altissima* [5]	2019	100%	1486.8 ± 194.3	78.3 ± 10.2	95% [42.4%, 51.0%, 6.6%]	5%
*Citrus limon* [5]	2019	0%	0	0.9 ± 0.6	0%	100%
*Crataegus monogyna* [5]	2019	0%	0	36. 7 ± 23.8	0%	100%
*Juglans regia* [5]	2019	60%	0.6 ± 0.3	1.4 ± 0.7	30% [100.0%, 0.0%, 0.0%]	70%
*Juniperus communis* [5]	2019	0%	0	0	-	-
*Pistacia lentiscus* [5]	2019	0%	0	0	-	-
*Quercus ilex* [5]	2019	60%	1.1 ± 0.4	2.6 ± 1.9	29% [62.5%, 0.0%, 37.5%]	71%
*Robinia pseudoacacia* [4]	2019	0%	0	1.6 ± 1.6	0%	100%
*Schinus mole* [5]	2019	0%	0	0	-	-
**47 days post-inoculation**						
*Ailanthus altissima* [5]	2019	100%	5441.4 ± 1437.4	286.4 ± 75.7	95% [54.8%, 44.4%, 0.8%]	5%
*Citrus limon* [5]	2019	0%	0	0	-	-
*Crataegus monogyna* [5]	2019	0%	0	0	-	-
*Juglans regia* [5]	2019	0%	0	0.2 ± 0.2	0%	100%
*Juniperus communis* [5]	2019	0%	0	0	-	-
*Pistacia lentiscus* [5]	2019	0%	0	0	-	-
*Quercus ilex* [5]	2019	0%	0	0.1 ± 0.1	0%	100%
*Robinia pseudoacacia* [4]	2019	0%	0	0	-	-
*Schinus mole* [5]	2019	0%	0	0	-	-
**61** **days post-inoculation**						
*Ailanthus altissima* [5] *Citrus limon* [5]	2019	100%	1588.3 ± 567.7	83.6 ± 29.9	95% [64.7%, 34.9%, 0.4%]	5%
	2019	0%	0	0	-	-
*Crataegus monogyna* [5]	2019	0%	0	0	-	-
*Juglans regia* [5]	2019	0%	0	0	-	-
*Juniperus communis* [5]	2019	0%	0	0	-	-
*Pistacia lentiscus* [5]	2019	0%	0	0	-	-
*Quercus ilex* [5]	2019	0%	0	0	-	-
*Robinia pseudoacacia* [4]	2019	0%	0	0	-	-
*Schinus mole* [5]	2019	0%	0	0	-	-

**Table A2:** Number of replicates for each plant species [N]; percentage of plants infested with live *Aculus mosoniensis*; number of live and dead *A. mosoniensis* per gram of plant biomass (mean ± SE) collected from each plant species tested; proportion of live and dead *A. mosoniensis*, and of adult females and males, and juveniles among the live *A. mosoniensis* collected from each plant species tested, at different sampling dates (i.e., 36 and 63 days post-inoculation, respectively) in 2020. (NA—data not available).

Plant Species [N]	Year	Plants with Live *Aculus mosoniensis*	*Aculus mosoniensis* Per Gram of Plant Biomass		Proportion of *Aculus mosoniensis*	
			Live	Dead	Live [Females, Males, Juveniles]	Dead
**36 days post-inoculation**						
*Ailanthus altissima* [5]	2020	100%	12,096.2 ± 2324.6	NA	NA [61.5%, 38.3%, 0.2%]	NA
*Azadirachta indica* [5]	2020	100%	194.6 ± 129.3	NA	NA [19.2%, 80.8%, 0.0%]	NA
*Corylus avellana* [5]	2020	80%	837.7 ± 474. 8	NA	NA [32.8%, 67.2%, 0.0%]	NA
*Olea europaea* [5]	2020	40%	2.6 ± 1.8	NA	NA [81.1%, 18.9%, 0.0%]	NA
*Rhus coriaria* [4]	2020	75%	272.0 ± 144.5	NA	NA [16.5%, 83.5%, 0.0%]	NA
*Vitis vinifera* [5]	2020	100%	5.1 ± 2.5	NA	NA [33.1%, 66.9%, 0.0%]	NA
**63** **days post-inoculation**						
*Ailanthus altissima* [5]	2020	100%	1378.5 ± 657.7	1765. 1 ± 353.9	45% [49.9%, 45.8%, 4.2%]	55%
*Azadirachta indica* [5]	2020	0%	0	0	-	-
*Corylus avellana* [5]	2020	0%	0	0	-	-
*Olea europaea* [5]	2020	40%	0.2 ± 0.1	0.2 ± 0.2	71% [60.0%, 40.0%, 0.0%]	29%
*Rhus coriaria* [4]	2020	25%	0.5 ± 0.5	0	100% [100.0%, 0.0%, 0.0%]	0%
*Vitis vinifera* [5]	2020	0%	0	0	-	-
